# Challenges to quality in contemporary, hybrid general practice a multi-site longitudinal case study

**DOI:** 10.3399/BJGP.2024.0184

**Published:** 2024-11-12

**Authors:** Rebecca Payne, Francesca Dakin, Ellen MacIver, Nadia Swann, Tabitha Pring, Aileen Clarke, Asli Kalin, Lucy Moore, Emma Ladds, Joseph Wherton, Sarah Rybczynska-Bunt, Laiba Husain, Nina Hemmings, Sietse Wieringa, Trisha Greenhalgh

**Affiliations:** Nuffield Department of Primary Care Health Sciences, University of Oxford, Oxford.; Nuffield Department of Primary Care Health Sciences, University of Oxford, Oxford.; Nuffield Department of Primary Care Health Sciences, University of Oxford, Oxford.; Nuffield Department of Primary Care Health Sciences, University of Oxford, Oxford.; Nuffield Department of Primary Care Health Sciences, University of Oxford, Oxford.; Nuffield Department of Primary Care Health Sciences, University of Oxford, Oxford.; Nuffield Department of Primary Care Health Sciences, University of Oxford, Oxford.; Nuffield Department of Primary Care Health Sciences, University of Oxford, Oxford.; Nuffield Department of Primary Care Health Sciences, University of Oxford, Oxford.; Nuffield Department of Primary Care Health Sciences, University of Oxford, Oxford.; Peninsula Schools of Medicine and Dentistry, University of Plymouth, Plymouth.; Nuffield Department of Primary Care Health Sciences, University of Oxford, Oxford.; Nuffield Trust, London.; Nuffield Department of Primary Care Health Sciences, University of Oxford, Oxford.; Nuffield Department of Primary Care Health Sciences, University of Oxford, Oxford.

**Keywords:** continuity, digital health, hybrid care, patient-centredness, quality, remote care

## Abstract

**Background:**

Since 2022, general practice has shifted from responding to the acute challenges of COVID-19 to restoring full services using a hybrid of remote, digital, and in-person care.

**Aim:**

To examine how quality domains are addressed in contemporary UK general practice.

**Design and setting:**

Multi-site, mostly qualitative longitudinal case study, placed in UK national policy context.

**Method:**

Data were collected from longitudinal ethnographic case studies of 12 general practices (2021–2023), multi-stakeholder workshops, stakeholder interviews, patient surveys, official reports, and publicly accessible patient experience data. Data were coded thematically and analysed using multiple theories of quality.

**Results:**

Quality efforts in UK general practice occur in the context of cumulative impacts of financial austerity, loss of resilience, increasingly complex patterns of illness and need, a diverse and fragmented workforce, material and digital infrastructure that is unfit for purpose, and physically distant and asynchronous ways of working. Providing the human elements of traditional general practice (such as relationship-based care, compassion, and support) is difficult and sometimes even impossible. Systems designed to increase efficiency have introduced new forms of inefficiency and have compromised other quality domains such as accessibility, patient-centredness, and equity. Long-term condition management varies in quality. Measures to mitigate digital exclusion (such as digital navigators) are welcome but do not compensate for extremes of structural disadvantage. Many staff are stressed and demoralised.

**Conclusion:**

Contemporary hybrid general practice features changes (digitalisation, physical distancing, extension of roles, and protocolisation) that have had the unintended effect of dehumanising, compromising, and fragmenting care. Policymakers and practices should urgently address the risks to patients and the traditional core values of general practice should be urgently addressed.

## Introduction

In 2020, primary care shifted to predominantly remote modalities as the COVID-19 pandemic took hold.^1^^–^^4^ A crisis-driven expansion of technology^5^ enabled digital booking, triage, and information exchange.^6^^,^^7^ Long-term condition (LTC) monitoring was deprioritised in the UK^8^^,^^9^ and internationally.^10^^,^^11^ In the UK, these changes occurred in the context of a decade of financial austerity,^12^ declining numbers of GPs,^13^ an ageing population with rising levels of multimorbidity,^14^^–^^16^ and increasing inequalities.^17^ Pressures on general practice in the period 2020–2023 were exacerbated initially by the acute crisis of the COVID-19 pandemic and then by a backlog of demand (partly linked to secondary care waiting lists),^18^ as well as a prevailing culture of austerity.^19^

By 2023, working practices had become hybrid (a mixture of in-person and remote),^20^ as digital modalities, such as video^21^^,^^22^ and e-consultations,^23^^–^^27^ and chatbots,^28^ were withdrawn, curtailed, or deferred. While there remains much academic and policy interest in digital technologies,^29^ most remote consultations and patient–practice messaging still occur by telephone.^30^ The authors of this study and others have explored the impact of these changes on isolated quality domains.^31^^–^^41^ But to our knowledge, no previous in-depth assessment has been published of the overall quality challenges facing contemporary hybrid general practice.

To address this gap, we asked: how have changes in technologies and working practices in UK general practice impacted — positively and negatively — on the various domains of quality?

**Table:** How this fits in

Quality in primary care is a multidimensional construct embracing effectiveness, efficiency, safety, patient-centredness, equity, continuity, accessibility, and more. We report on how UK practices have striven to deliver on these aspects of quality as they move to a hybrid model that combines in-person with remote and digital care. The context for quality is currently very challenging, with resource constraints, staff shortages, and weak infrastructure. Digital systems intended to increase efficiency have produced some benefits for some people but have created new forms of inefficiency, increased fragmentation of care, contributed to staff stress, and widened inequities of access.

## Method

### Origin of this sub-study

The work was part of Remote by Default 2 (RBD2), for which the protocol,^42^ baseline findings,^30^ and other sub-studies^32^^,^^33^^,^^36^^,^^38^ have been published. The main project is a multi-site 28-month case study of 12 UK general practices across England, Scotland, and Wales (2021–2023). Practices were purposively sampled to represent a wide range of digital maturity levels from traditional (few digital services) to digital leaders (providing state-of-the-art digital services and supporting other practices). Using an adapted researcher-in-residence model, one member of the research team built a relationship with practice staff, made repeated ethnographic visits, conducted interviews (in-person or remotely), and collected descriptive statistics.^43^ Longitudinal data were synthesised to build a picture of how the introduction of remote and digital services was evolving.

Additional work packages included key informant interviews and multi-sector workshops with clinical, policy, industry, and citizen stakeholders, and co-design work with patients and staff to develop more patient-centred pathways for digital access.

Early data from RBD2 revealed quality concerns (for example, whether patients’ clinical and relational needs were being met) and trade-offs (such as digital access for some achieved at the expense of digital exclusion for others). We therefore set out to create a subset of data on key dimensions of quality for targeted analysis.

### Theoretical framework

We combined three established quality frameworks, summarised in Box 1,^34^^,^^35^^,^^44^^–^^46^ whose domains mapped well to our emerging data. Our analysis also incorporated theoretical insights from sociological^47^ and socio-technical literature.^48^^,^^49^

**Box 1.** Domains of quality investigated in this study

**Institute of Medicine Framework (6 domains)^44^** **Safety**: Avoiding harm to patients from the care that is intended to help them **Efficacy**: Providing services based on scientific knowledge to all who could benefit and refraining from providing services to those not likely to benefit (avoiding underuse and misuse, respectively) **Patient-centredness**: Providing care that is respectful of and responsive to individual patient preferences, needs, and values and ensuring that patient values guide all clinical decisions **Timeliness**: Reducing waits and sometimes harmful delays for both those who receive and those who give care **Efficiency**: Avoiding waste, including waste of equipment, supplies, ideas, and energy **Equity**: Providing care that does not vary in quality because of personal characteristics such as sex or gender, ethnicity, geographic location, and socioeconomic status **Starfield’s Primary Care Assessment Tool (4 primary domains)^45^^,^^46^** **First-contact, accessible care**, undifferentiated by disease modality, for all aspects of health except emergencies **Comprehensive**, whole-patient care (going beyond reactive management of health problems on demand and including prevention and attention to the social determinants of health) **Coordination**, both in primary care services and at the interfaces with other parts of the healthcare system **Continuity** of care over time **Ladds *et al*’s four domains of continuity in primary care^34^^,^^35^** Continuity of the **relationship** with a single clinician over time Continuity of **distributed work** among a **multidisciplinary team** Continuity of **illness episodes** (same clinician from first presentation to resolution or transfer of a problem) Continuity of **commitment to the practice population** (measured, for example, in terms of length of service in the same practice)

### Creating a focused dataset covering quality domains

**Figure 1.**
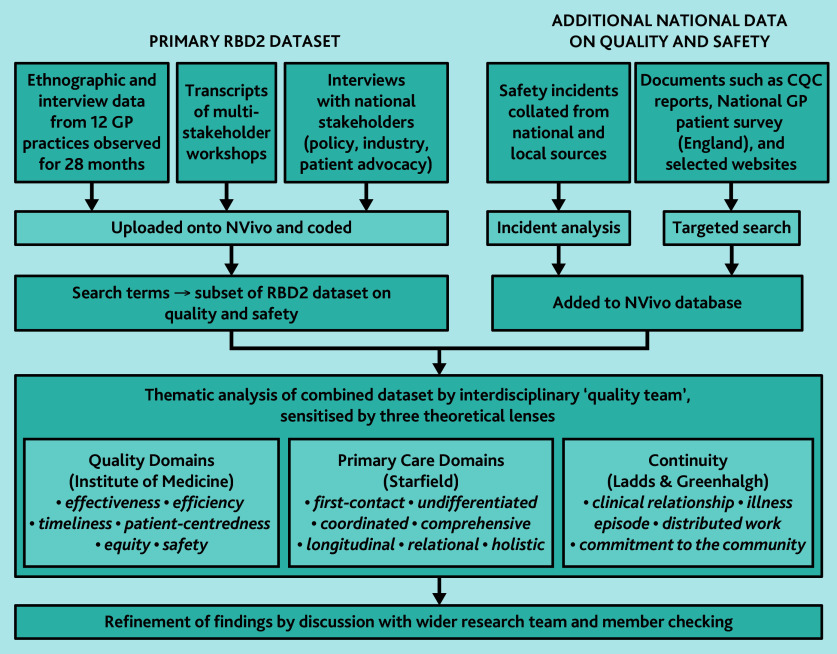
Sources of data and approach taken to analysis.

To obtain a subset of practice-level data, we shared the Institute of Medicine framework, Primary Care Assessment Tool, and continuity frameworks with the RBD2 researchers-in-residence and asked them to highlight relevant interviews and observations from their fieldwork. We searched the full RBD2 dataset (including workshop transcripts) using keywords derived from the quality domains in Box 1, and obtained practice-level data from the English GP Patient survey 2023^50^ (which covered eight of the 12 participating practices). We also downloaded and analysed patient reviews (127 in total) posted on the eight English RBD2 practice websites. To obtain additional national-level data on quality issues, we selected extracts from our stakeholder interviews and workshops, and obtained official reports.^51^^–^^53^ Data were collated into a single RBD2 subset on quality. This process is illustrated in Figure 1.

These data sources and their contribution and caveats are summarised in Table 1.

**Table 1.** Data sources and contribution

**Source, type of data, dates**	**Description of full dataset**	**Subset of data analysed for this article**	**Contribution and caveats of this data source**
Multi-site longitudinal case study of remote care in general practice (September 2021–December 2023)^42^	12 general practices (eight in England, two in Wales, two in Scotland) followed for 28 months. Ethnography, staff and patient interviews, and documents (annual reports, websites, leaflets). Data transcribed and coded in NVivo	Extracts from field notes, interviews, and documents in which quality domains (Box 1) were mentioned (∼150 pages of extracts)	In-depth ethnographic material providing rich insights into the functioning and priorities of modern UK general practice, covering a key 2-year period as practices transitioned to the ‘new normal’
Stakeholder interviews (2021–2023)^42^	Stakeholders in strategic roles at national and local level in England, Wales, and Scotland sampled from policy (arm’s length bodies, government, health boards), industry, training providers, and patient advocacy	Extracts from these interviews which covered key quality domains (∼120 pages total)	‘Bird’s eye view’ provided by senior stakeholders and experts from across the UK, main emphasis on policymakers but also includes other sectors
GP Patient Survey for England (2023)^50^	National survey of patients in English general practice, conducted by NHS England	Summary of findings on quality for practices across England 2021–2023	Large and rigorous survey conducted annually. Limited to England and to specific questions asked
Online reviews by patients (2021–2023)	209 online patient reviews from eight general practices in England, hosted on NHS practice websites (comparable data were not available in Wales or Scotland)	Extracts relevant to quality domains	Unedited dataset containing patient opinion and experiences of care. Unverifiable; may be biased towards poor experiences
Multi-stakeholder workshop on quality and safety (September 2022)^42^	Online workshop with clinicians, national clinical leads, representatives from arm’s length bodies, practice staff, and lay people (*n* = 61). Plenaries and breakout groups recorded on video	Interdisciplinary discussions on quality. Video footage totalling 4 hours, transcribed into 11 pages of extracts	Diverse and nuanced discussions among a large number of participants from various sectors. Breakout groups facilitated the capture of a wide range of perspectives
English CQC state of care reports (2021–2022^51^ and 2022–2023),^52^ plus inspection reports on two RBD2 practices	Annual assessment of the state of health and social care in England by the independent regulator. Health component covers specific questions in five domains: access to care, quality of care delivered, inequalities, workforce, and systems. Individual CQC inspections of two RBD2 practices	Extracts relevant to quality of care in general practice nationally and to the wider context in which care is delivered. Specific data on two participating practices	Data gathered from a wide range of inspection activity and internally validated by CQC. Limited to England. Data relate to areas of interest to CQC; they were collected for a particular purpose (regulation and formal monitoring)
Healthcare Safety Investigations Body report on continuity of care and delayed diagnosis (2023)^53^	Official investigation into impact of continuity of care on time to diagnosis	Full report	In-depth report analysing the impact of poor continuity on safety in English general practice. Based on a single case and England only

*CQC = Care Quality Commission.*

### Data management and analysis

Data were uploaded to NVivo v14 and explored thematically with reference to the quality domains in Box 1.^54^ The analysis team (three academic GPs, a public health academic, a social scientist, a patient advocate, and a medical student) used a five-step process: close reading to gain familiarity; identifying themes using the criterion ‘captures something important in relation to the overall research question’;^54^ discussion among pairs of researchers to share initial interpretations and resolve differences through dialogue; selecting illustrative excerpts; and themes and examples presented to the wider team.

For example, one major category in the dataset (part of the theme of ‘context’) was ‘workforce’. Within that, there were several sub-categories, including stress among support staff, which recurred across interviews from all 12 practices and was sometimes mentioned repeatedly in the same interview. Support staff talked of their own stress and that of their colleagues; clinicians and patients acknowledged stress in support staff. Through close reading and discussion, and by returning to the data (including looking for disconfirming examples), we were able to identify factors contributing to support staff stress, link these data with quality domains such as coordination and patient safety, and explore how practices tried to reduce stress among their staff.

An initial set of draft findings was shared with the wider research team, along with contacts from participating practices and our external advisory group, and refined in response to their input (see Acknowledgements for details).

## Results

### Outline

The rich and multi-source (mostly qualitative) dataset collected over 28 months provided insights into two overarching themes: first, the overall context for quality in contemporary UK general practice (which included various demographic and policy-driven changes over and above digitalisation) and second, the challenges encountered by the 12 participating general practices as they strove to deliver on quality in this changing context, which involved balancing the trade-offs between different dimensions of quality. As summarised in Box 2, each overarching theme included several categories. All categories were evident to some extent in all participating practices regardless of their level of digital maturity; detailed comparisons between the practices will be reported separately. All practice names are pseudonyms.

**Box 2.** Summary of key themes in findings

**Theme 1: The context for quality** Increasing complexity and a system under strain Financial austerity and a loss of resilience across the system More complex patterns of illness and need Changing workforce and changing roles Infrastructure under strain Loss of in-person interactions among staff **Theme 2: Delivering on quality (balancing benefits and trade-offs of hybrid services)** Care and compassion versus efficient transactions Access and triage: matching supply with need and demand Long-term conditions: protocolised versus personalised care Addressing equity and inclusivity: mitigating the digital inverse care law

### The context for quality

#### Increasing complexity and a system under strain

Our combined data sources, including our empirical data and grey literature (Table 1), revealed a vastly changed and changing context. In the past 5 years, the complexity of general practice — clinical, technical, and operational — has greatly increased, with ever more components, actors, health conditions, and wider needs, technologies, stressors, and demands.

#### Financial austerity and loss of resilience across the system

Our period of data collection occurred during an extended period of government policy oriented to reducing public spending. Qualitative data from participating practices, along with official reports,^51^^–^^53^ revealed numerous examples of under-resourced and withdrawn services, leading to bottlenecks, blocks, and tensions at the interfaces between services. Secondary care services had tightened referral criteria, and introduced required pre-referral work-ups and trials of therapy. Secondary care provided ‘advice and guidance’ instead of seeing a patient, or saw them once only and asked the GP to follow them up; patients who missed their secondary care appointments were discharged. This GP voices the frustration:

*‘I know that the hospital isn’t twiddling their thumbs. I know they’re busy. I know they’ve never been busier. But we certainly have a frustration that work is dumped on us, but it’s secondary* [care*] guys’ responsibility.’*

(GP, Westerly)

When GPs sought to push back against such ‘shifted’ work, practice staff had to contact secondary care and explain decisions to patients. Likewise, loss of resilience in the social care sector (reduced overall funding, reduced capacity, closure of some services, and longer waits for assessment) and within wider social networks (reduced informal community support) meant that practice teams had to spend time signposting to, form-filling for, or compensating for gaps. As well as being time consuming, this was challenging because of lack of knowledge, training, and shared records.

#### More complex patterns of illness and need

Staff in many participating practices reported that the population they served was getting older, sicker, and more socially isolated, as well as consulting more frequently. Our study was not designed to verify these impressions quantitatively.

#### A changing workforce and changing roles

Our interviews and ethnographic observations revealed reports of high staff turnover, perceived unmet training needs, and high levels of stress and sickness absence (covered in more detail in separate articles ^33^^,^^38^). Senior receptionists confided that newly recruited support staff, in particular, sometimes became disillusioned and left quickly as the full demands of the role became apparent. In some practices, there were unfilled posts for GPs (or insufficient funds to employ additional qualified GPs). Stress on partners in particular was sometimes exacerbated by a high burden of supervision of other multidisciplinary team (MDT) members.^52^^,^^53^ GPs in some practices had shifted to seeing only the most complex cases, eroding continuity and creating decision fatigue.^53^

#### Infrastructure under strain

While novel digital technologies were evident in some practices, digital infrastructure sometimes lacked the required functionality, speed, or capacity, resulting in systems that were, in the words of one staff member, *‘not up to the job’* (Practice Manager, Fernleigh). Limitations in how information was stored and (re) presented sometimes made it difficult for MDT members to follow the trajectory of a patient’s illness or understand others’ contributions.^53^ Expansion in numbers of staff had intensified pressure on physical space (some staff described taking their administrative work home because there was nowhere to work).

Telephone systems were frequently a source of frustration. Patients often spent a long time trying to get through; not all systems made it clear that the patient was working their way up a queue; and calls were sometimes dropped after a fixed period of waiting. Some practices had subcontracted telephone answering to call centres, leading to an impersonal experience for patients.

Purchasing decisions made at locality level sometimes left practices with no choice but to use technologies that were unfit for purpose, with standardised procurement processes and cost pressures resulting in a limited choice of technologies or key functionality being excluded from the specifications. Some practices procured their own technologies, but this could prove challenging in terms of the specialised knowledge and skills required.

#### Loss of in-person interactions among staff

It was evident from our ethnographic visits that digital technologies allowed both individual and collaborative work to happen without staff being physically co-located or working synchronously in time. Some staff welcomed improved options for interacting with other MDT members, especially those (such as pharmacists) who were not physically co-located. However, even when staff were working in the same building, emails and instant messages often replaced telephone calls or spontaneous in-person conversations (such as when people passed in the corridor or met in the staff room), reducing real-time interactions between staff and thus the opportunity to build strong relationships.

### Delivering on quality (balancing benefits and trade-offs of hybrid services)

Our dataset included many positive comments from staff and patients about the increased convenience and flexibility of a hybrid service. This was evident in ethnography, interviews, and many of the patient reviews:

*‘The option of telephone really cheesed a lot of people off [initially], “how can I be … treated over the phone?” and things like that. But I think gradually … patients have got used to that and a lot of patients are actually liking the convenience of not having to attend the GP and wait in the waiting room and can just have a quick call.’*

(Receptionist, Westerly)

Some staff talked about improved data capture from patient self-monitoring:

*‘So she actually put in the blood pressure readings [on FootFall] that she’s done at home because she had a big list of them. So it means she can go away now and do that herself, and that I’ll upload on to our system on to her records … And the good thing with the blood pressure is it actually gives us the average return at the bottom. … So that’s been good.’*

(Healthcare assistant, River Rd)

However, there were also numerous, sometimes troubling, examples of quality in one domain achieved at the expense of compromised quality in (an)other domain(s). In the quote below, a GP talks about possible improvements to patient safety and variety of consultation modalities achieved at the expense of increased workload:

*‘So electronic system*[s]*, that’s the big key* [thing] *is patient safety, risk management … but not workload. It’s increased workload. And you know, it’s meeting other needs and it’s giving us other capacity, other tools to help consult the patients. So there are benefits, but not in terms of capacity. It’s reduced our capacity.’*

(GP, River Rd)

Such trade-offs were evident across all the RBD2 practices, regardless of digital maturity, suggesting that they may be inherent. Below, we offer four illustrative examples of key quality trade-offs that were evident in our data.

#### Example 1: Care and compassion versus efficient transactions

Patients’ highest priorities involved the human and relational aspects of general practice including familiarity, warmth, empathy, compassion, and effective communication, as the following quote illustrates:

*‘I’ve been with this practice for nearly 10 years, along with my kids. I am really happy with them. The doctors listen to me, share their expertise, make me feel involved, and give me choices about my care. The nurses are friendly and professional, and put us at ease, even with scary things like jabs for the kids. Reception are always very kind, explain things clearly, give useful options for appointment times, and don’t make me feel rushed.’*

(Online patient review, Ogden East, February 2023)

Our ethnographic findings showed that both clinicians and support staff drew on their relational knowledge and human qualities to adapt care to patients’ needs. They often knew, for example, when a person lived alone or had a disabled relative. Some practices had a ‘usual doctor’ or ‘buddy group’ system to optimise relational continuity and this was highly valued by patients; others used a ‘most appropriate clinician’ arrangement, reducing relational continuity (as we have reported in more detail elsewhere^35^).

Despite the contextual pressures described in the previous section, staff in participating practices were strongly committed to delivering the human elements of quality (captured in the quality domains in Box 1 as patient-centredness, whole-patient care, equity, and relational continuity), and expressed frustration when they were unable to:

*‘If somebody rings and they’re expecting [a baby] and they’re bleeding, we’re signposting them straight to the early pregnancy assessment unit but it’s pressure … and I just think that if somebody is expecting and they’re bleeding, I just think it’s nicer to come from the GP.’*

(Receptionist, Fernleigh)

The receptionist in the above quote articulates a crucial tension — between what high-quality general practice would look like in an ideal system (the patient’s own, known GP would witness their suffering and guide them through the personal crisis of potential early pregnancy loss) and the transactionally framed compromises that are currently happening because of ‘pressure’. Note, however, that this was a staff member’s perspective; the patient may have been very happy to omit the GP step in this illness journey.

#### Example 2: Access and triage: matching supply with need and demand

All RBD2 practices had some kind of triage system designed to allocate patients to the most appropriate staff member and prioritise needy and urgent patients. These systems varied considerably, both in terms of technologies (including apps and web forms, usually with the facility for sending attachments, automated telephony systems, and receptionists asking questions of the patients, often from a flowchart or pro forma) and in terms of who did the triaging (receptionist, back-office support staff, pharmacist, nurse, allied health professional, GP trainee, or established GP), where it was done (designated triage room, variable office space, clinical room, home), how the patient was contacted (email, telephone call-back, text message), how other members of the MDT were informed (intranet messages, direct entry on to record, shared lists, paper artefacts such as sticky notes), and what rules and heuristics were used (for example, in one practice, a patient was allowed to book an in-person appointment only if the triage doctor allowed it).

Despite these differences, demand for general practice care often (although not always) exceeded supply. Practice staff made strong arguments that if demand went unchecked, systems would quickly become overwhelmed, leading to urgent problems and vulnerable patients being lost in the sheer volume of requests. In the quote below, a pharmacist reflects on how the introduction of electronic consultations led to a deluge of requests for minor (and usually self-limiting) problems:

*‘I think to be honest with you, e-Consult has really left a scar on the practice [laughs] because at first it sounded great, you know, we had this system that was going to triage all these patients, and whatever, but I think the problem was it was just there … it was being abused … and perhaps, yes, we do need to educate patients to explain what the system is, how it should be used, you know.’*

(Pharmacist, Caerleon)

Some support staff described being able, through triage, to offer on-the-day appointments to people judged as needing them, and some clinicians gave examples of time freed up to spend with complex patients. But digital triage systems also had significant unintended consequences across the quality domains (Box 3) — so much so that some practices had begun to discontinue or limit their use.

**Box 3.** Unintended consequences of remote and digital triage

**Loss of organisational efficiency** Skilled staff members were needed to do the triaging, reducing capacity elsewhere in the system. If unskilled staff were used, patients sometimes ended up seeing the wrong person Pre-assessment questions generated large amounts of information (much of it irrelevant) that staff did not have time to read Patients were not always available when called back. Sometimes practices cancelled patients after two failed call-backs, requiring re-booking Over-protocolised work processes led to double-handling (for example, if an annual review did not include reauthorisation of medication, the patient needed a second appointment) Triaging software and formalised flowcharts usually required patients to prioritise one problem, so a patient with multiple problems had to submit multiple requests and see multiple people. A GP could usually deal holistically (and more efficiently) with all the problems Some algorithms were risk-averse, misclassifying patients as emergencies when they knew they were not (possibly resulting in a flawed instruction to ‘dial 999’ or ‘go to A&E’), while others lacked granularity (sending patients to services such as optometry or pharmacy which were inappropriate to their needs) When triage was subcontracted to a call centre, lack of local knowledge (such as which nurse does the baby jabs) could lead to patients booked in to see the wrong clinician Patients sometimes spent a proportion of the consultation complaining about how hard it was to access services, and staff spent time explaining or apologising In practices where web forms were used as a common pathway for all patients, support staff time was spent converting telephone or walk-up requests into web format The rapid pace and high risks associated with triage increased staff stress, contributing to burnout, sickness absence, and staff turnover **Loss of accessibility** Many patients found the digital triage system time consuming, difficult, and stressful to navigate Some less digitally enabled patients, including those from vulnerable groups (such as those who are older, sicker, or who had more complex needs) experienced the digital front door as impassable (although some, including patients with hearing impairments, welcomed text-based interaction) Some practices did not proactively inform patients about new ways of accessing the practice (such as via an app) because they were already overwhelmed by existing demand **Loss of timeliness** While some patients were fast-tracked, others experienced delayed care **Loss of patient-centredness** Assessment by structured symptom checklist and decision algorithm was impersonal and disheartening, as well as time consuming Call-backs occurred at inappropriate times (for example, when in a meeting, teaching a class, or driving a bus) **Inequities** Disadvantaged groups (see Box 4) had particular problems navigating the system ‘Gaming’ of web forms by patients with high health and digital literacy to achieve a desired outcome (for example, an in-person appointment with a doctor) meant there were even fewer slots available for less enabled patients **Threats to safety** Because of low patient health literacy, ‘red flag’ symptoms (such as acute chest pain) occasionally ended up in a ‘next working day’ queue for attention **Loss of continuity** In most practices, digital triage meant the patient was less likely to see the same doctor for repeated appointments

**Box 4.** Challenges to equity in remote and digital general practice

**Structural and poverty-related factors** Geographical variation in broadband availability and strength (some remote areas had limited service) No (or entry-level) digital devices Low data package Device(s) shared among several family members No private space in the home No home Weak social networks (nobody to help or support) **Capability factors** Low health literacy, digital literacy, or system literacy Inability to read or understand text Cognitive conditions (such as learning difficulties, brain fog, memory loss) Neurodiversity Reflexivity and mastery (for example, the ability to assess a social situation and plan accordingly) Visual or hearing impairment Low fluency or confidence in the language used in the practice

#### Example 3: Long-term conditions: protocolised versus personalised care

Most RBD2 practices suspended routine LTC monitoring during the pandemic; many took some time (in one case, 3 years) to re-start. Practices varied in how they did LTC reviews; some had mostly reinstated in-person appointments. Others used structured web-based forms completed by the patient or a support staff member (with the patient either present in person or on the phone), automatically populating the Quality and Outcomes Framework template, which triggered payment. LTC reviews could thus be marked as completed without seeing or speaking to the patient.

Staff raised concerns that while web forms could capture data on patients who had previously defaulted from LTC reviews, patients did not always fill them out accurately. Patients sometimes failed to declare symptoms either because of low health literacy (for example, assuming cough and breathlessness to be normal in asthma) or not wanting to bother the practice. In some cases, staff doubted that patients were competent in using the devices they had been supplied with; some suspected that certain patients invented the readings.

Nurses expressed concern that heart failure reviews (mostly in older patients) conducted by telephone or web form were likely missing some cases of deterioration because a key component of assessment was a visual overview of the patient’s general condition (such as speed of walking, breathlessness at rest, or swollen ankles). The in-person assessment had also been an opportunity to reinforce patient education through conversations held in the context of an existing, ongoing therapeutic relationship. Nurses worried that not all patients would be able to learn what they needed to know from leaflets sent as email attachments or links to web resources.

Algorithmic, remote management of LTCs is built on the implicit assumption that all symptoms in a particular organ system are a result of the known LTC. Worsening respiratory symptoms in patients with chronic obstructive pulmonary disease, for example, could trigger a prescription for steroids and antibiotics on the assumption that this indicates an exacerbation, but the opportunity to examine a patient and detect an early lung cancer will be missed. Another concern was that some patients who would benefit from in-person assessment on clinical grounds might insist on a telephone appointment on convenience grounds.

Variability in quality of care for diabetes was a particular concern of practice staff and national stakeholders. This new, life-changing diagnosis was sometimes delivered by telephone, making it difficult to establish the therapeutic relationship on which ongoing care could be built. Most practices conducted interim reviews by telephone and some even did annual reviews this way, raising major concerns about omission of key examinations (such as of the feet or insulin injection sites). In one or two practices, annual reviews were now mostly completed by healthcare assistants and with most information supplied by the patient in advance; practice nurses spent much less time with patients so were less able to gain a holistic perspective and answer patients’ questions. Positive changes to diabetes care included the use of pharmacists to review complex drug regimens, group consultations which provided opportunities for peer support and vicarious learning, and (in some practices) a reallocation of clinician time to undertaking visits to housebound patients.

The Care Quality Commission (CQC) has raised concerns about clinical quality and equity in LTC management, commenting that insufficient support is provided, especially to patients from minority ethnic groups.^51^^,^^52^ Both CQC and patients expressed concerns about annual LTC reviews for people with autism and learning difficulties; problems included the review not happening at all, carer not invited or included, and patient not being offered an in-person appointment, thereby missing a visually obvious deterioration.

#### Example 4: Equity and inclusivity: mitigating the digital inverse care law

Like all UK public services, general practices are required to accommodate people with variable digital set-up and capability.^55^ Box 4 lists the challenges to equity that were evident in the RBD2 practices. Some of these (such as poor broadband and widespread poverty) were beyond practices’ direct control, while others (such as how well patients could read) were not always known.

Intermediation roles to help patients use technology — variously termed digital navigator, support worker, and patient liaison officer — were used increasingly by practices as our fieldwork progressed. These individuals were usually reception staff who had learned new digital navigation skills on the job,^33^ and were performing this new role in addition to their traditional one. While these individuals were often perceived as helpful, their dual role meant a high workload, high cognitive demands, and consequently increased stress. A single patient with complex needs could take a significant proportion of the time available (one interviewee described helping a patient navigate the complex online benefits system). In practices where most patients had markers of disadvantage, the need for digital navigator support significantly exceeded supply.

### Theorisation

Socio-technical theorists remind us that technologies are not merely tools that we use for certain tasks.^48^^,^^49^^,^^56^ Rather, *‘work practices are conceptualized as networks of people, tools, organizational routines, documents and so forth’*.^56^ Technologies, and their affordances (the things we can and cannot do with them), constitute our work and change who we are in the workplace. If LTC management occurs using symptom checklists and data fields populated remotely by the patient, for example, this aspect of care becomes impersonal and transactional, and the person checking those data becomes a data processor.

Several phenomena observed in this study — persistence of various remote and (especially) digitally mediated interactions, subcontracting the digital front door of the practice to a call centre, use of monitoring protocols which require the patient to submit data for subsequent (semi-automated) processing by support staff, and a tendency for staff to interact asynchronously — can be analysed through a sociological lens. These profoundly changed ways of working are examples of two wider phenomena originally described by Giddens back in the 1980s: distanciation,^57^ defined as ‘[t] he stretching of social systems across time–space’^47^ and disembedding,^58^ in which social activities occur increasingly at a distance, ‘removed from the immediacies of context’^47^ on the basis of technologically mediated and abstracted forms of information.

Distanciation and disembedding replace the warmth of a here-and-now, in-person interaction with words on a screen, tick-boxes, algorithmic pathways, and mounting task lists. Social interactions, instead of being contextually grounded and richly meaningful, become ‘emptied out’, dehumanised, and deprofessionalised.^47^ The example above of reception staff signposting a patient with early pregnancy bleed directly to a specialist diagnostic unit epitomises this shift. In this and similar examples, the very essence of a GP’s expertise — as a clinical generalist, an expert in the patient as a person, a professional witness to suffering, and someone who accompanies the patient on their illness journey — has disappeared from the aspects of care that are being acknowledged, measured, and reimbursed.

As complexity theory predicts, the multiple elements of quality and their various interdependencies generate emergent tensions and paradoxes that are not amenable to simple fixes but which require creative responses and ongoing adaptation.^59^

## Discussion

### Summary

This longitudinal, qualitative study of 12 UK general practices, along with stakeholder interviews and data from workshops, official reports, and patient experience, supplemented by national-level documents, has produced five principal findings, which draw together a number of themes and categories in the data.

First, the current context for delivering quality in UK general practice is characterised by accumulated financial austerity, loss of resilience, increasingly complex patterns of illness and need, an increasingly fragmented workforce, material and digital infrastructure that is unfit for purpose, and fewer in-person interactions.

Second, while most clinicians and support staff continue to aspire to the traditional values of general practice (relationship-based, holistic, compassionate care, and ongoing support to patients and families), providing the human elements of care is increasingly challenging.

Third, we have revealed an important paradox: digital access and triage systems and multiple new staff roles designed to increase efficiency appear to have introduced multiple new forms of inefficiency while compromising other domains of quality including accessibility, patient-centredness, and equity.

Fourth, the quality of LTC management varies. While some practices have reintroduced traditional in-person reviews, others rely on remote, asynchronous data entry by patients and fragmented care shared between clinically qualified staff and assistants with limited training.

Finally, measures to improve equity and mitigate digital exclusion (such as digital navigators) have been introduced and are helping to some extent, but they do not compensate for the complexity of systems and extremes of structural disadvantage.

Overall, these findings reveal a system that is approaching — or, in some cases, beyond — breaking point. Staff members are stressed, demoralised, and leaving; clinical care appears to be compromised; and many patients are dissatisfied, frustrated, and unable or less willing to seek care. We believe there are significant risks to patient safety and to the future survival of traditional general practice in UK.

### Strengths and limitations

We believe this is the first study to examine a full range of structures and processes in hybrid general practice and their impact on quality. By combining multiple methods and data sources, and applying established quality frameworks and theoretical lenses, we were able to describe and examine how quality is achieved or why it is not achieved in different settings. The researcher-in-residence model allowed team members to develop a deep knowledge about their linked practice, and regular research meetings fostered reflection and discussion of themes and categories across the 12 practices. The study was almost entirely qualitative, so our findings need to be interpreted alongside more quantitative designs^39^^,^^40^ and official statistics.^50^^,^^60^ While we incorporated selected findings from such sources into our case studies, we chose not to use publicly available Quality and Outcomes Framework data for the eight English practices because of the limited granularity of this source. Although the RBD2 practices were located in England, Scotland, and Wales, several of the additional data sets such as the CQC reports and GP patient survey related to England only.

### Comparison with existing literature

This study confirms earlier research which has set out a challenging context for UK primary care including austerity,^19^ task shifting from secondary care,^61^ weakening of the social care sector,^62^ rising consultation rates, especially in relation to multimorbidity, poverty-related stress, and mental health conditions,^14^^,^^20^^,^^63^ and a backlog of routine work following the acute phase of the pandemic.^11^ Our empirical findings on staff shortages resonate with wider evidence that the general practice workforce has undergone numerous and substantial changes.^31^^,^^60^ Many GP partners have retired or left the profession, to be replaced, in part, by salaried GPs, locums, and allied and associate roles,^60^ facilitated by the Additional Roles Reimbursement Scheme (introduced in 2019 and subsequently expanded in England,^64^^–^^66^ with some similar contractual changes in Scotland).^67^ Our findings on infrastructure are borne out by national reports, which have highlighted that general practice infrastructure in the UK (both material and technological) is increasingly unfit for purpose.^53^^,^^68^^,^^69^

Earlier studies have also demonstrated that digital access models may reduce rather than increase efficiency.^6^^,^^27^^,^^70^ Our findings align with previous work that has demonstrated challenges to quality when primary care services are digitised, including clinical effectiveness,^27^ safety,^27^^,^^32^^,^^71^^,^^72^ training,^33^ continuity,^34^^,^^35^ access and equity,^36^^,^^37^ prescribing,^73^^,^^74^ preventive medicine,^75^ LTC management,^76^ staff workload and wellbeing,^38^^,^^77^^,^^78^ and that loss of continuity is associated with reduction in efficiency (Kajaria-Montag H, *et al*, unpublished data, 2024). The current study also aligns with recent publications highlighting the impact of social isolation,^79^ how the cost of living crisis is reducing health,^80^ and with evidence showing that quality across a range of domains is impacted when GP numbers fall, even when other healthcare professionals fill their place.^13^

### Implications for practice

Digitalisation, care at a distance, expansion of roles, protocolisation, and other recent changes intended to improve general practice services have had the unintended effect of compromising quality as traditionally defined. While the picture is not universally bleak, and while staff members continue to do their best to deliver high-quality, compassionate care under difficult circumstances, there is evidence of systematic erosion of the less measurable and more humanistic elements of ‘quality’ general practice.

Policy solutions must take account of prevailing realities including resource constraints and workforce shortages. But policy needs to engage with more than the question of what the latest ‘efficiency fix’ (structural, technological, or otherwise) should be. A great strength of traditional UK general practice was the values-driven ethos, which celebrated the personal, holistic, relationship-based, and longitudinal nature of the ‘family doctor’ system. Staff who worked and trained in such a system imbibed these values, became part of a community of practice, and passed the values on to the next generation. However, if staff are embedded in a fragmented, physically distanced, transactional, depersonalised system that is continually reacting to overwhelming demand and struggling with burnout, that system is what they will learn and reproduce. Those designing policy solutions must, therefore, ask what urgent interventions can be mobilised to retain and strengthen the core elements of primary care (Box 1) that underpin a quality service. We are currently working with educators, clinicians and policymakers to apply our findings in a way that reduces staff stress and improves the patient experience and outcomes.
